# Atraumatic Shoulder Dislocation Revealing a Brain Tumor: A Case Report

**DOI:** 10.7759/cureus.98472

**Published:** 2025-12-04

**Authors:** Kazuhiro Ikeda, Shotaro Teruya, Hiromitsu Tsuge, Ryunosuke Watanabe, Shinzo Onishi

**Affiliations:** 1 Department of Orthopedic Surgery, Institute of Medicine, University of Tsukuba, Tsukuba, JPN

**Keywords:** astrocytoma, brain tumor, epileptic seizure, seizure-related injury, shoulder dislocation

## Abstract

Non-traumatic shoulder dislocation may occasionally result from seizure-related disorders, and, in rare cases, from underlying brain tumors. We report a 23-year-old man who presented with anterior shoulder dislocation without apparent trauma. Computed tomography revealed a large Hill-Sachs lesion, a bony Bankart lesion, and a coracoid process fracture - findings inconsistent with a low-energy mechanism of injury. Because the injury occurred during sleep, an epileptic seizure was suspected. Further neurological evaluation identified focal epilepsy originating from the right frontoparietal region, and brain magnetic resonance imaging revealed a tumor in the right frontal lobe. Surgical resection confirmed an isocitrate dehydrogenase-mutant, Central Nervous System World Health Organization grade 2 astrocytoma. Following tumor resection and antiseizure therapy, the patient remained seizure-free while continuing conservative management for shoulder instability. Both the bony Bankart lesion and the coracoid process fracture had achieved bony union, the American Shoulder and Elbow Surgeons score reached 100, and functional recovery was excellent.

This case highlights the importance of considering seizure-related or intracranial pathology in atraumatic shoulder dislocation and underscores the pivotal role of orthopedic surgeons in the early recognition of underlying central nervous system disease.

## Introduction

Shoulder dislocation accounts for approximately 50%-60% of all joint dislocations [1], and most commonly results from traumatic events, including sports injuries or falls [1,2]. In contrast, non-traumatic shoulder dislocations are uncommon and may occasionally result from underlying central nervous system disorders, including epileptic seizures [3]. In such cases, dislocation often occurs during an unwitnessed seizure, and patients usually present to orthopedic clinics without an apparent history of trauma [4]. As a result, clinical attention tends to focus exclusively on local joint pathology, making it difficult to identify the underlying neurological cause. Because seizure control is closely linked to orthopedic outcomes - particularly the risk of recurrent dislocation [5,6] - orthopedic surgeons must remain vigilant for undiagnosed epileptic seizures.

We present a rare case of astrocytoma that initially manifested as an atraumatic anterior shoulder dislocation caused by an unrecognized epileptic seizure. At the initial evaluation, the orthopedic surgeon suspected a seizure based on the atypical injury mechanism and imaging findings, which led to the timely diagnosis of an underlying astrocytoma. The objective of this report is to highlight key historical and radiographic features that may prompt orthopedic surgeons to consider seizure-related or intracranial pathology in cases of atraumatic shoulder dislocation.

## Case presentation

A 23-year-old man without any prior medical history or medications was brought to our hospital with severe left shoulder pain and restricted mobility. He reported no apparent traumatic event and stated that he awoke with sudden, intense pain in his left shoulder. As he lived alone, there were no witnesses at the time of onset. The emergency physician diagnosed a left anterior shoulder dislocation and performed manual reduction. Six days after the injury, the patient visited our Orthopedic Department. The shoulder was immobilized in internal rotation using a sling and swathe. He had no relevant medical history, and this was his first occurrence of shoulder dislocation. The Beighton score [7] was 0 out of 9, indicating no generalized joint laxity.

**Figure 1:**
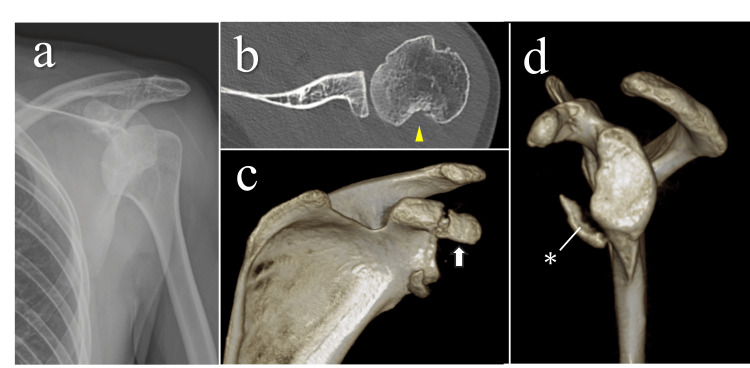
Radiological findings at the time of dislocation and after reduction (a) Anteroposterior radiographs showing anterior shoulder dislocation. (b) Axial CT image demonstrating a Hill-Sachs lesion (20 mm wide and 12 mm deep; yellow arrowhead). (c) Three-dimensional CT image showing a coracoid process fracture classified as Type II according to Ogawa’s classification (white arrow). (d) Three-dimensional CT lateral view showing a bony Bankart lesion involving approximately 20% of the glenoid rim (asterisk). CT, computed tomography

Computed tomography (CT) revealed a relatively large Hill-Sachs lesion (Figure 1), a bony Bankart lesion, and a coracoid process fracture, classified as Type II according to Ogawa’s classification [8]. These findings suggested that the shoulder dislocation had occurred with relatively high energy. The fact that the injury occurred during sleep, combined with these imaging findings, raised suspicion of an epileptic seizure. The patient was, therefore, referred to the Neurosurgery Department for further evaluation. To prioritize evaluation and management of the underlying neurological condition, we opted for conservative treatment of the shoulder at that stage. After the neurological condition was stabilized, we electively planned surgical stabilization.

**Figure 2:**
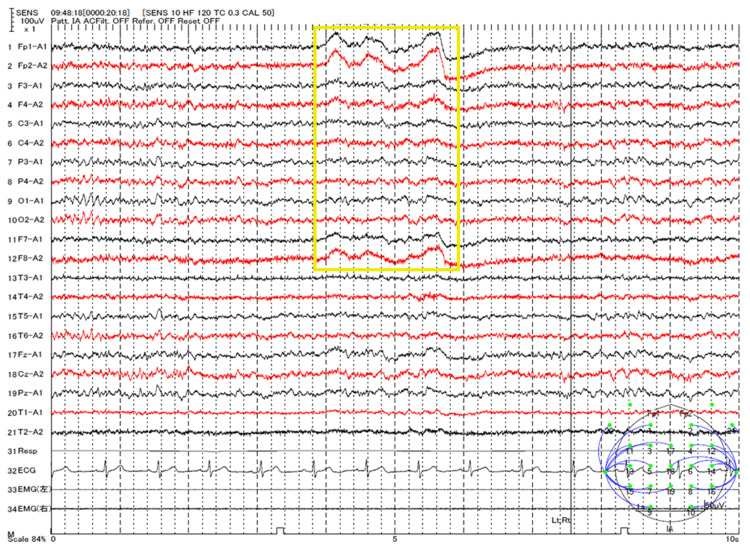
Electroencephalogram findings Electroencephalogram revealed sharp and spike-and-slow wave complexes in the right frontocentral to parietal regions (channels 1-13, yellow box), indicating epileptiform discharges originating from the same area during the interictal period.

Three weeks after the injury, the patient was referred to the Neurosurgery Department. He reported that, during the past six months, he had experienced three to four episodes of severe back pain upon awakening, which left him unable to get out of bed. He also recalled associated episodes of urinary incontinence and tongue biting. Together, these symptoms suggested unrecognized nocturnal seizures. An electroencephalogram revealed focal epilepsy originating from the right frontoparietal region (Figure 2). Brain magnetic resonance imaging (MRI) was scheduled, and oral lacosamide was initiated at a dose of 200 mg per day.

Four weeks after the initial injury, the patient experienced another epileptic seizure during sleep, resulting in recurrent dislocation of the left shoulder. The shoulder was manually reduced in the Emergency Department.

**Figure 3:**
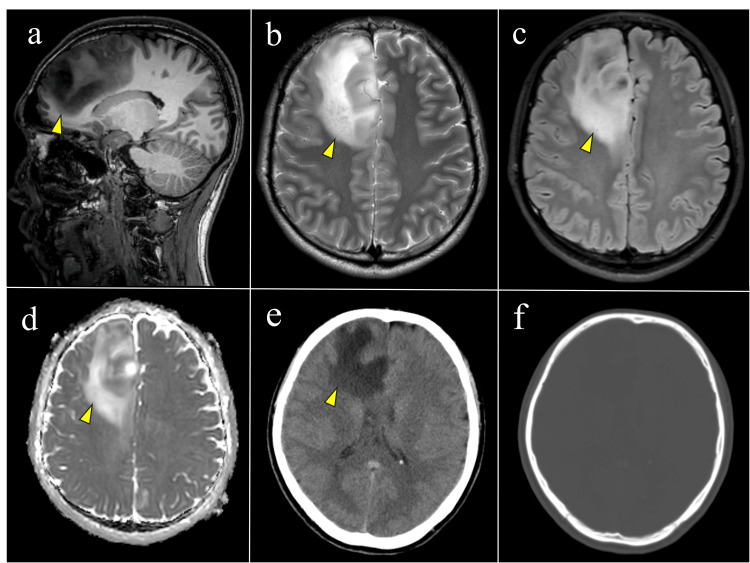
Brain MRI and CT findings (a) Sagittal T1-weighted image showing a hypointense lesion in the deep white matter of the right frontal lobe (yellow arrowhead). (b) Axial T2-weighted image and (c) axial FLAIR image showing a hyperintense lesion with an ill-defined margin. (d) ADC map showing no restricted diffusion in the same region. (e) Non-contrast CT showing a low-density area in the right frontal white matter (yellow arrowhead) without evidence of hemorrhage. (f) Bone-window CT confirming the absence of calcification. MRI, magnetic resonance imaging; FLAIR, fluid-attenuated inversion recovery; ADC, apparent diffusion coefficient; CT, computed tomography

Five weeks after the injury, brain MRI revealed a poorly demarcated lesion in the deep white matter of the right frontal lobe, without calcification on CT, suggestive of a neoplastic process (Figure 3). Astrocytoma was considered the most likely diagnosis, and surgical resection of the tumor was scheduled.

**Figure 4:**
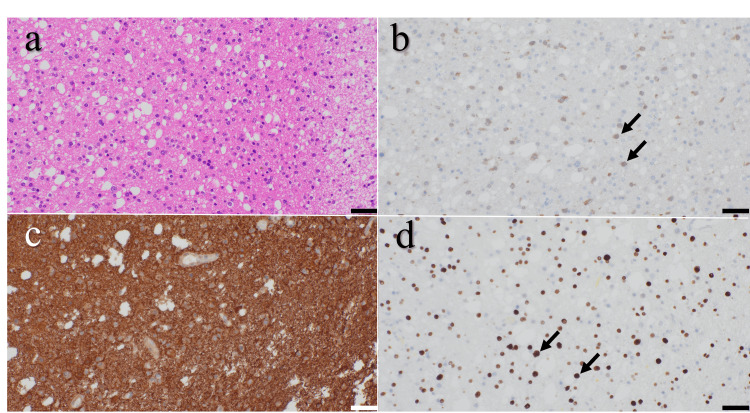
Histopathological findings (a) Hematoxylin and eosin staining showing increased cellularity with nuclear pleomorphism, consistent with a glial neoplasm. (b) Immunohistochemical staining for IDH1 showing diffuse cytoplasmic positivity (arrows), indicating the presence of an IDH1 p.R132H mutation. (c) GFAP staining showing strong and diffuse positivity in tumor cells, consistent with astrocytic differentiation. (d) Olig2 staining showing nuclear positivity in tumor cells (arrows). These findings are consistent with astrocytoma, IDH-mutant, CNS WHO grade 2, and the scale bar represents 50 μm. IDH, isocitrate dehydrogenase; GFAP, glial fibrillary acidic protein; CNS WHO grade, Central Nervous System World Health Organization grade

Two months after the initial injury, craniotomy and tumor resection were performed, achieving approximately 90% subtotal removal. Postoperatively, the patient experienced transient limitation of left shoulder elevation, which recovered within several weeks, leaving no persistent motor or higher brain dysfunction. Histopathological examination confirmed an isocitrate dehydrogenase (IDH)-mutant astrocytoma, Central Nervous System World Health Organization (CNS WHO) grade 2 (Figure 4). Given the patient’s young age, low-grade histology, and minimal residual tumor, adjuvant therapy was not administered, and a wait-and-scan strategy was adopted. The patient received oral perampanel (4 mg/day) for four months postoperatively for seizure control.

Three months after the initial injury (one month postoperatively), the patient revisited our department. The range of motion of the shoulders was as follows: forward flexion, 180°/160°; abduction, 180°/160°; external rotation at the side, 70°/55°; external rotation at 90° abduction, 90°/75°; and internal rotation to the T5/T7 vertebral level. He reported mild pain at the end range of motion. Because the patient’s willingness to undergo surgery had decreased, we decided to continue conservative management.

**Figure 5:**
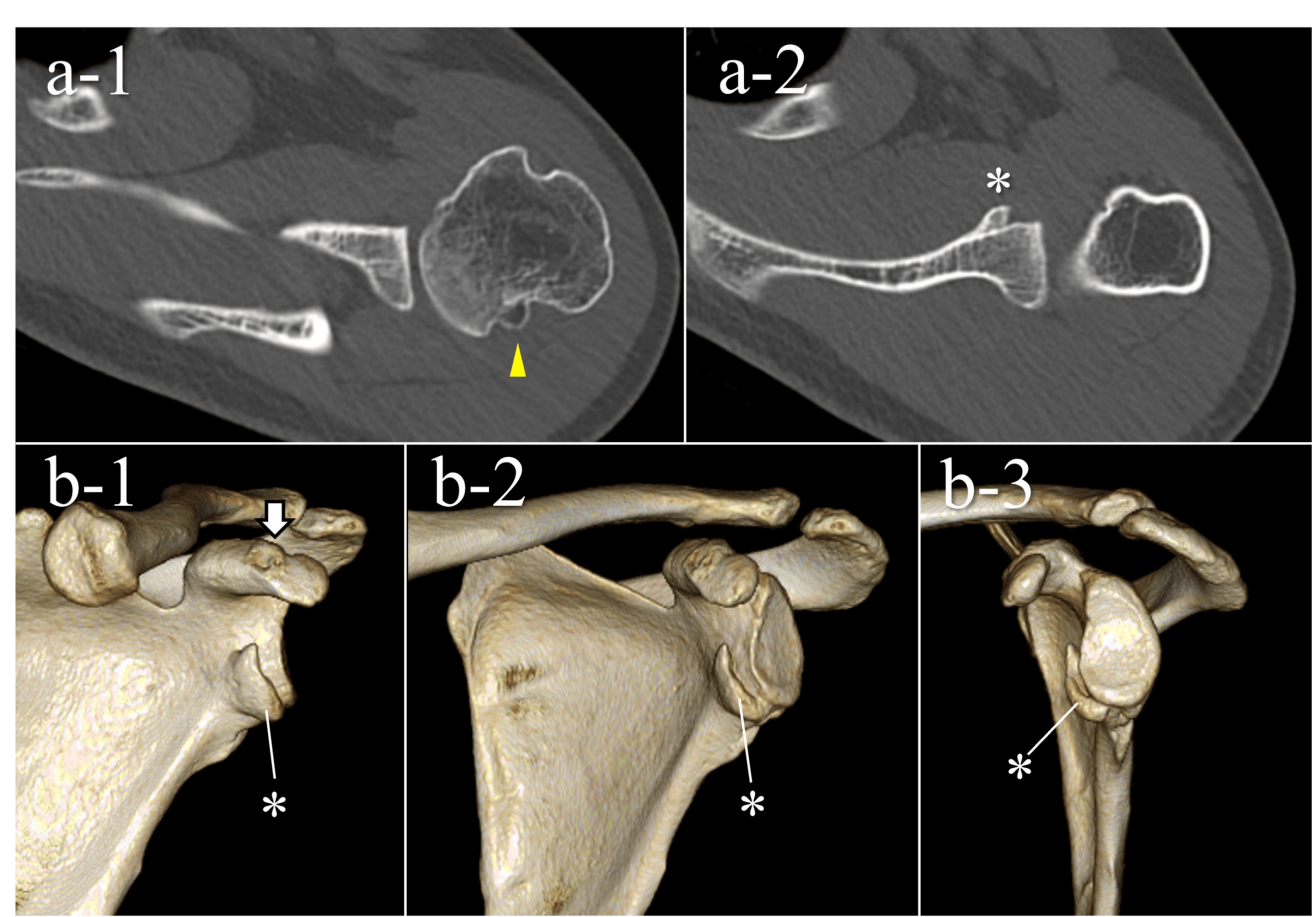
CT findings at one year after injury Axial CT images show bony union of the separated fragment within the Hill-Sachs lesion (a-1, arrowhead) and healing of the bony Bankart lesion deep inside the glenoid (a-2, asterisk). Three-dimensional CT reconstructions demonstrate bony union of the coracoid process (b-1, arrow) and the bony Bankart lesion (b-1,2,3, asterisk). CT, computed tomography

One year after the injury (seven months postoperatively), there was no recurrence of epileptic seizures or shoulder dislocation. The range of motion of the shoulder was as follows: forward flexion, 180°/165°; abduction, 180°/165°; external rotation at the side, 70°/70°; external rotation at 90° abduction, 90°/85°; and internal rotation to the T5/T5 vertebral level. The patient had no pain or limitations in activities of daily living, and the American Shoulder and Elbow Surgeons (ASES) score was 100. CT images demonstrated bony union of both the coracoid process fracture and the bony Bankart lesion (Figure 5). As the patient did not wish to undergo further surgery, long-term follow-up was planned.

## Discussion

This case represents an example in which an astrocytoma was incidentally discovered following a shoulder dislocation caused by an epileptic seizure. The key clinical implication of this case is the early suspicion of a seizure-related disorder by the orthopedic surgeon, which facilitated the timely diagnosis of the underlying brain tumor.

This case differs from typical shoulder dislocations in that there was no apparent traumatic event. When a primary shoulder dislocation occurs during sleep or rest without an identifiable injury mechanism, an epileptic seizure should be strongly suspected [3]. Because seizures may occur without witnesses or patient awareness, clinicians should thoroughly explore subtle historical clues through detailed questioning. During history taking, clinicians should ask about the presence of witnesses, limb convulsions, tongue biting, urinary incontinence, and postictal confusion [9]. In epilepsy, lateral tongue biting is a highly specific finding, with a reported specificity of 96%, and is therefore an important diagnostic clue [10]. Postictal myalgia upon awakening also served as a valuable clue in differentiating nocturnal seizures.

Parasomnias, including rapid eye movement sleep behavior disorder, should also be considered as a differential diagnosis for shoulder dislocation occurring during sleep. Such conditions can be distinguished from epileptic seizures by the absence of confusion, tongue biting, or urinary incontinence, and by normal electroencephalographic findings [11]. Collaboration with neurologists or sleep specialists may be required in shoulder dislocations occurring during sleep.

In addition, epileptic seizures can often be suspected based on local shoulder findings. Seizure-related dislocation occurs as a result of tonic muscle contractions, and posterior dislocation tends to develop due to the strong contraction of the adductor and internal rotator muscle groups [12]. Although posterior shoulder dislocation is a rare injury, accounting for only 2%-5% of all shoulder dislocations [13], it represents 36%-50% of dislocations associated with epileptic seizures [3,14]. Therefore, in cases of posterior shoulder dislocation without a clear traumatic event, an epileptic seizure should raise a strong suspicion. Furthermore, anterior shoulder dislocations associated with epileptic seizures have been reported to involve relatively large Hill-Sachs and bony Bankart lesions [3]. These findings were consistent with our case. Such extensive bony lesions are thought to result from sustained, forceful muscle contractions during epileptic seizures, generating significant compressive and shear forces on the articular surface [3]. A comprehensive assessment of the patient’s history and imaging findings enabled the consideration of an epileptic seizure as a differential diagnosis at the initial orthopedic visit.

IDH-mutant grade 2 glioma has been reported to have a five-year survival rate of 97.6%, even in cases of subtotal resection without adjuvant therapy [15,16]. Therefore, a relatively favorable prognosis can be expected in this case. Reconstructive surgery should be staged with attention to tumor control and neurological stability, especially in young and active patients. Since epileptic patients have been reported to have a threefold higher risk of redislocation after shoulder stabilization compared with non-epileptic individuals [6], seizure control plays a crucial role in determining surgical outcomes. A noteworthy consideration is that perioperative stress and surgical invasion have been reported to worsen seizure control in a subset of patients, making careful timing and multidisciplinary perioperative management essential [17]. In line with general epilepsy management principles, it is desirable to confirm a seizure-free period of at least six months before elective shoulder stabilization whenever feasible [18,19]. The patient has remained seizure-free since neurosurgical resection and is now considered a suitable candidate for shoulder stabilization surgery. However, bone union was achieved, and functional stability in daily activities was restored through conservative management. This favorable outcome underscores the importance of careful nonoperative treatment and close collaboration with neurosurgery in seizure-related shoulder dislocations. A longer follow-up is needed to confirm sustained shoulder stability in this case.

## Conclusions

We reported a case of astrocytoma presenting with shoulder dislocation. Early recognition of a seizure during the initial orthopedic visit contributed to timely diagnosis and improved prognosis. In shoulder dislocations without a clear traumatic mechanism, a detailed history should be taken with attention to seizure-related or central nervous system disorders. Seizure-related shoulder dislocation may present with characteristic imaging features, including posterior dislocation, as well as relatively large Hill-Sachs and bony Bankart lesions in anterior dislocations. Orthopedic surgeons should carefully interpret fracture and dislocation patterns in the context of the injury mechanism and imaging findings to identify potential underlying conditions.
